# Rapid multi-dynamic algorithm for gray image analysis of the stroma percentage on colorectal cancer

**DOI:** 10.7150/jca.58887

**Published:** 2021-06-01

**Authors:** Tengfei Li, Zekuan Yu, Yan Yang, Zhongmao Fu, Ziang Chen, Qi Li, Kundong Zhang, Zai Luo, Zhengjun Qiu, Chen Huang

**Affiliations:** 1 Department of Gastrointestinal Surgery, Shanghai General Hospital, Shanghai Jiao Tong University, Shanghai 201600, China.; 2 Graduate School of Bengbu Medical College, Bengbu 233000, China.; 3 Academy for Engineering and Technology, Fudan University, Shanghai 200433, China.; 4 Key Laboratory of Industrial Dust Prevention and Control & Occupational Health and Safety, Ministry of Education.; 5 School of Medical Instrument and Food Engineering, University of Shanghai for Science and Technology, Shanghai 200093, China.; 6 Department of Medical Oncology, Shuguang Hospital, Shanghai University of Traditional Chinese Medicine, Shanghai 200021, China.

**Keywords:** Colorectal cancer, Tumor stroma percentage, Rapid multi-dynamic, Threshold algorithm, Gary image

## Abstract

**Background:** Tumor stroma percentage (TSP), as an independent, low-cost prognostic factor, could complement current pathology and act as a more feasible risk factor for prognosis. However, TSP hadn't been applied into TNM staging. Here, the objective of our study was to investigate the prognostic significance of TSP in a robust rapid multi-dynamic approach with the application of MATLAB and threshold Algorithm for Gray Image analysis.

**Methods:** Using a retrospective collection of 1539 CRC patients comprising three independent cohorts; one SGH cohort (N=996) and two validation cohorts (N =106, N= 437) from 2 institutions. We investigated 996 CRC of no special type. According to our established thresholds, 357 cases (35.84%) were classified as TSP-high and 639 cases (64.16%) as TSP-low. We determined the gray image area as the stromal part of the WSI and calculated the stroma percentage with our proposed method on MATLAB software.

**Results:** In both TSP-cad(50%) and TSP-cad(median), multivariate analysis showed the TSP-cad was an independent prognostic factor for the vessel invasion and tumor location. For OS, TSP-manual HR=1.512 (95% CI 1.045-2.187); TSP-cad HR=1.443 (95% CI 0.993-2.097) and TSP-cad(median) HR=1.632 (95% CI 1.105-2.410). Fortunately, TSP-manual and TSP-cad were also found independent prognostic factor in all the cohorts. It was found that TSP-cad had slightly higher HR and wider CI than TSP-manual.

**Conclusions:** Our research showed that TSP was an independent prognostic factor in CRC. Moreover, threshold algorithm for the quantitation of TSP could be established. In conclusion, with this Rapid multi-dynamic threshold Algorithm for Gray Image counting of TSP, which showed a higher accuracy than manual evaluation by pathologists and could be a practical method for CRC to guide clinical decision making.

## Introduction

Colorectal cancer (CRC) is the third most common malignancy worldwide and the third leading cause of cancer-related mortality. The latest cancer statistics of the United States in 2020 showed that the estimated new cases of CRC accounted for nine percent in male population with cancer and eight percent in female with cancer. Overall, for all the solid tumors, CRC was ranked the forth in the morbidity and the second in mortality 1, in which roughly one fourth of the patients were diagnosed with stage II cancer. According to the tumor-node-metastasis (TNM) system of the American Joint Committee on Cancer (AJCC) classification, histological subtype was commonly applied in the staging of CRC 2. However, the TNM system was proved insufficient to predict the prognosis of patients with stage II CRC 3. There were some limitations in this classification since prognosis of patients in the same stage varied greatly 4,5. NIH guidelines recommend chemotherapy for potential high-risk stage II CRC, actually 5-year relapse-free survival (RFS) ranged from 44% to 83% in the stage III CRC 6-8. Due to early diagnosis and treatment, the mortality rate of CRC was declining. Pathologists usually visually evaluated tumor stroma percentage (TSP) on hematoxylin-eosin (H&E) stained sections under a microscope 9. There was still an increasing awareness of the clinical importance of evaluating TSP, also known as tumor stroma ratio (TSR) on postoperative H&E-stained sections 10. It was crucial to optimize risk stratification through personalized treatment to prevent undertreatment and overtreatment. The computer-aided detection (CAD) systems had been developed to quantify stroma percentage. Similar methods using Deep Learning CAD in CRC have shown better predictive power than expert human visual assessment 11. Therefore, there is an urgent need to apply semiautomatic software to rapidly analyze a large number of pathological sections and to complete current TNM staging in order to predict the prognosis of tumor patients.

Nowadays, it has been well acknowledged that the occurrence, growth and development of tumor is depended on the tumor microenvironment, and the tumor stromal is an indispensable part of the tumor microenvironment 12. Recently, attention has been attached to TSP's promising potential role in the prognosis of various tumor types. Therefore, TSP has become an important prognostic indicator for different tumor types 13-16.

Based on tumor-associated stroma, a promising prognostic parameter is the TSP. Previous studies further confirmed that TSP, as an independent, low-cost prognostic factor, was capable of completing current pathology which was reported as a more feasible risk factor for prognosis. The TSP was a prognostic tool that stratified tumors into TSP-low and TSP-high based on the quantity of stroma percentage in H&E-stained sections of specimens and it was proved to be a strong and independent prognostic parameter 10,17. It was shown that high stroma tumor was of poor prognosis in CRC as well as in other solid epithelial tumors 18-21, whereas low stroma tumor was predicted with a more favorable outcome. So far, manual visual assessment, including open source software semi-automatic assessment, was commonly used for evaluating the stroma percentage in clinic 11,22,23. Previous studies shown that TSP now could be evaluated by manual visual and semi-automatic methods 11,14,24,25. Tumor area was manually annotated in H&E-stained whole slide images (WSI) and thus a digital image feature of pathology was constructed. Quantitative features were extracted and reduced from the selected patches of tumor cell dense area 26. Furthermore, a deep learning-based algorithm could perform automated TSP assessment of the CRC subclass of rectum adenocarcinomas by the developed CNN (Convolutional Neural Networks) 11,27.

Unfortunately, the quantification of stroma percentage through manual visual assessment was mainly depended on the intra- and inter-observer variability. Only a small portion of malignant tumors were evaluated, which weren't capable of representing all the malignant tumors. Their overall accuracy in judging the stroma percentage was often limited. Besides, assessment of TSP wasn't widely adopted as a prognostic variable in CRC due to its lack of standardization. Therefore, we need a reliable semi-automatic method to enhance the competence of current treatment strategies to predict TSP in CRC. Based on supervised machine learning and pixel classification, a rapid multi-dynamic threshold algorithm which included image-based semi-automatic approach with open source software MATLAB (R2018b (V9.5); https://www.mathworks.com/), was built, trained and validated by using H&E-stained sections of specimens recently. This method had obtained good results by using robust rapid multi-dynamic algorithm.

By applying our semi-automatic method to select areas from whole-slide scanning images, it is ultimately intended to investigate and quantify the rapid multi-dynamic algorithm approaches in the stroma percentage of CRC stratified by the TSP, which may have increased prognostic significance of TSP.

## Materials and methods

### Patients' materials

The Shanghai General Hospital (SGH) cohort (N=996) were identified from a retrospectively collected database of patients undergoing surgery for CRC at the Shanghai General Hospital between 2014 and 2018. None of the patients had received neoadjuvant chemotherapy or radiotherapy and no mortality within 30 days of surgery. Furthermore, without formal colonic or rectal resection, patients who received endoscopy treatment were excluded from the research. Patients were staged clinically according to the 8th edition of American Joint Committee (AJCC) TNM classification. Clinicopathological characteristics were diagnosed and confirmed by two independent pathologists according to the guidelines of the AJCC on Cancer, and were presented in Table 1. Written informed consent was obtained from each patient before enrolling in the study. The study was approved by the Ethical Committee for Clinical Research of Shanghai General Hospital. We used computer-generated random numbers to assign 703 patients to the training cohort and 293 patients to the testing cohort. Besides, for the internal validation Tissue Microarray (TMA) cohort, an additional 106 paired CRC were collected from patients diagnosed with CRC at the General Surgery Department of Shanghai General Hospital from 2013 to 2014. All specimens, to construct the TMA, were paraffin-embedded, validated by H&E staining, and finally examined by two independent pathologists. For the external validation, The Cancer Genome Atlas (TCGA) cohort, an additional 437 patients with CRC stage I-Ⅳ were selected from the TCGA dates (https://portal.gdc.cancer.gov/). Two independent validation cohort of 543 consecutive patients were included using the same criteria as those of the SGH cohort to validate the predictive performance of the cohort. Detailed characteristics of patients for TMA cohort and TCGA cohort could be found in Supplementary Table 1 and Supplementary Table 2, respectively.

### H&E-stained sections scanning and imaging

**Figure 1:**
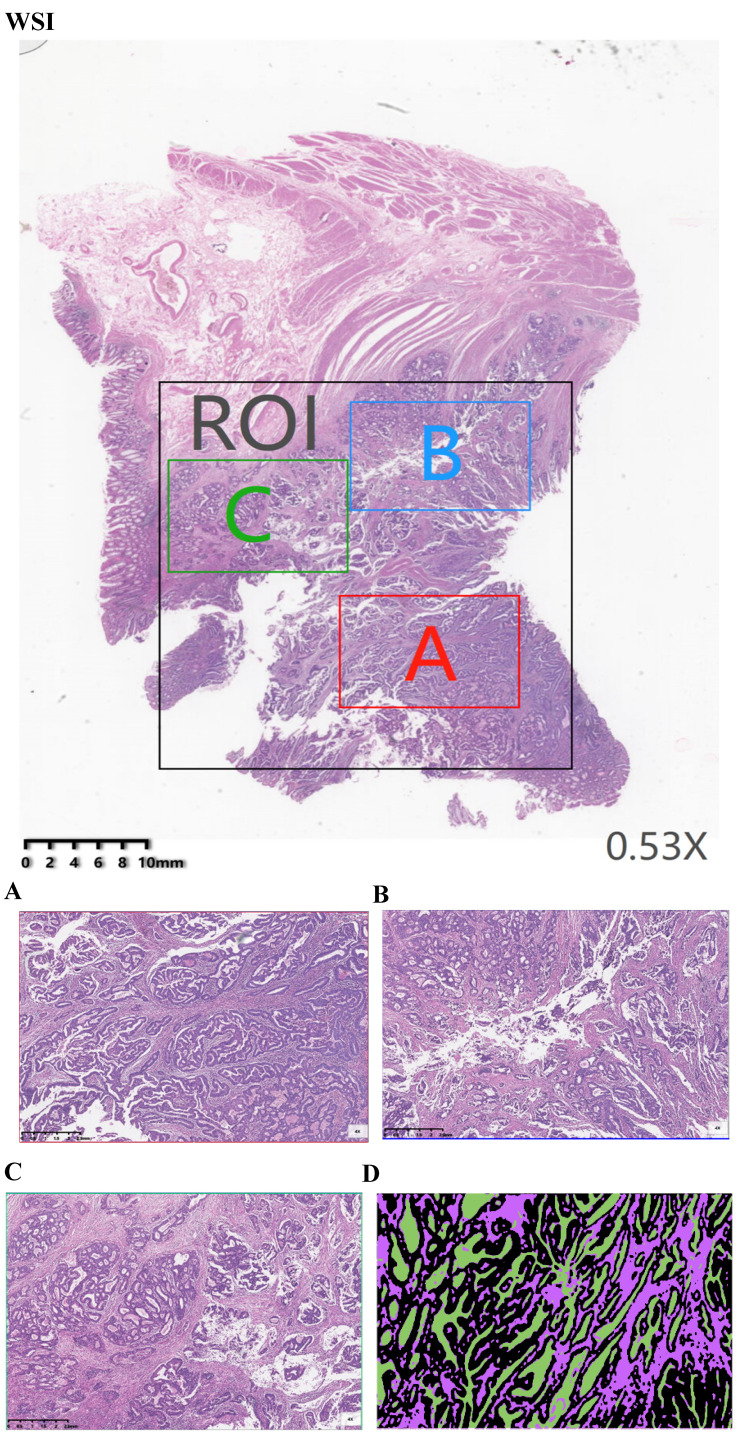
Example of a manual assessment at the tumor level. (WSI) Pathology signature construction in hematoxylin and eosin stained whole slide images. ROI, Area was annotated by a pathologist highlighting the region of interest. (A) Red annotation is the most invasive part. (B) Blue annotation. (C) Green annotation. (D) images were annotated using Black-tumor, Purple-stroma and Green-necrosis. A: red annotation, is the most invasive part; B: blue annotation; C: green annotation; Black: tumor; Purple: stroma; Green: necrosis.

H&E-stained sections were scanned using KF-PRO series automatic digital slice scanning system under 10× magnification. Each raw scanning whole-slide image (WSI) was annotated by a pathologist highlighting the region of interest (ROI) (Fig. 1WSI) using the digital slice reading software K-Viewer (Konfoong Biotech, NB, China, 1.5.3.1), which included the tumor and stroma. This software could be employed here to view the scanning images and to realize image zooming, annotation, tagging and other functions, which was compatible with local computers and networked mobile reading modes. From each whole-slide scanning image, three smaller images were extracted from the ROI. These annotations were adopted to calculate the map area for comparing the proportion of tumor and stroma in each section by using the open source image process software MATLAB. Therefore, three area representative of the tumor invasive fields of 1496 ×996 pixels (40× field) were selected (Fig. 1A, B and C). An area was examined under ×4 magnification to ensure that tumor cells was presented for an all-round view. For the multiple available parts, it was to give out the score of each part and to compute the average. A total of 2298 images were extracted from 996 WSIs. Each extracted image of the 40× fields was based on the annotations of an experienced pathologist on H&E-stained sections. Next, the selected imaging was analyzed by MATLAB. Since whole-slide scanning images contained various cells/tissues (e.g. fat, muscle, lymphocyte infiltrations, necrosis, healthy epithelium, stroma, erythrocytes, tumor, etc.), a semi-automatic learning algorithm was developed to segment the tissue (“Stroma”, “Tumor” and “Necrosis”) based on 40× fields of tumors originating from different patients. Therefore, the 2298 images were annotated using tumor, stroma and necrosis labels (Fig. 1D).

### Manual evaluation of the TSP

The primary tumors of both cohorts were scored for TSP on 5 μm H&E-stained tissue sections as described previously^10^,^19^. The tissue samples selected were those were defined as the most invasive part of the primary tumors as used by the pathologists to determine the T-status (Fig. 1A). To determine the TSP, the region with the highest stroma was selected using an 10× objective. A microscopy field was scored where tumor cells were presented at all borders of the image field (north, south, east, west) of the 4× objective. In case of tumor heterogeneity, each image field score was given by ten-fold of the scoring percentage in which the lowest one was selected as the final scoring percentage.

All slides were first scanned and digitized using the K-Viewer with the ×10 objective. Then, a representative area showing the most invasive part at low magnification (×4 objective) was selected. Subsequently, a single area, surrounded by tumor cells, in which both stroma and tumor existed at high magnification (×40 objective) was chosen. Despite some heterogeneity in the TSP among biopsy tissue blocks throughout the entire slide, the regions with the largest amount of stroma and the worst differentiation were selected as the representative object for analysis according to the previous study. Tissues that contained mucin or necrosis in the selected field were visually excluded. The interrater reliability was evaluated.

**Figure 2:**
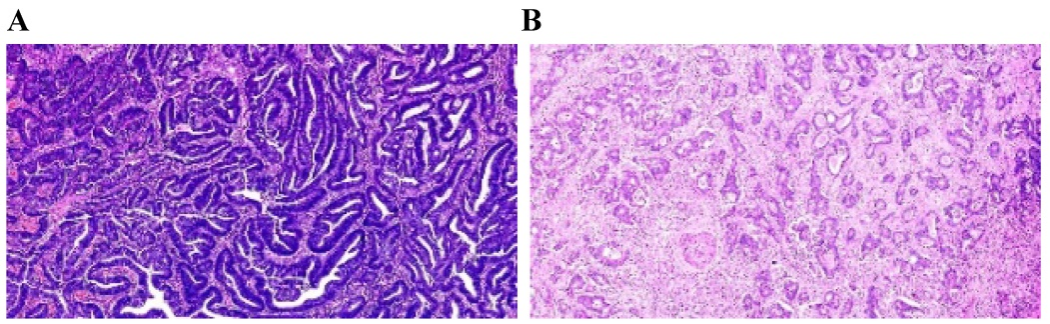
Manual visually assessment TSP. (A) TSP-manual^a^=20%≤50% was categorized as TSP-low with long OS. (B)TSP-manual^a^=70%>50% was regarded as TSP-high with short OS. Pathologist^a^ consensus.

The TSP was visually calculated (per tenfold: 10%, 20%, 30% and so forth) per field. In this study, it was considered that the tumor and stroma proportions were complementary. For example, TSP 70% represented that stroma accounted for 70% of the entire tumor tissue, and tumor cells accounted for 30%. In this study, a TSP ≤50% was categorized as TSP-low (Fig. 2A), and a TSP >50% was regarded as TSP-high (Fig. 2B).

### CAD evaluation of the TSP

**Figure 3:**
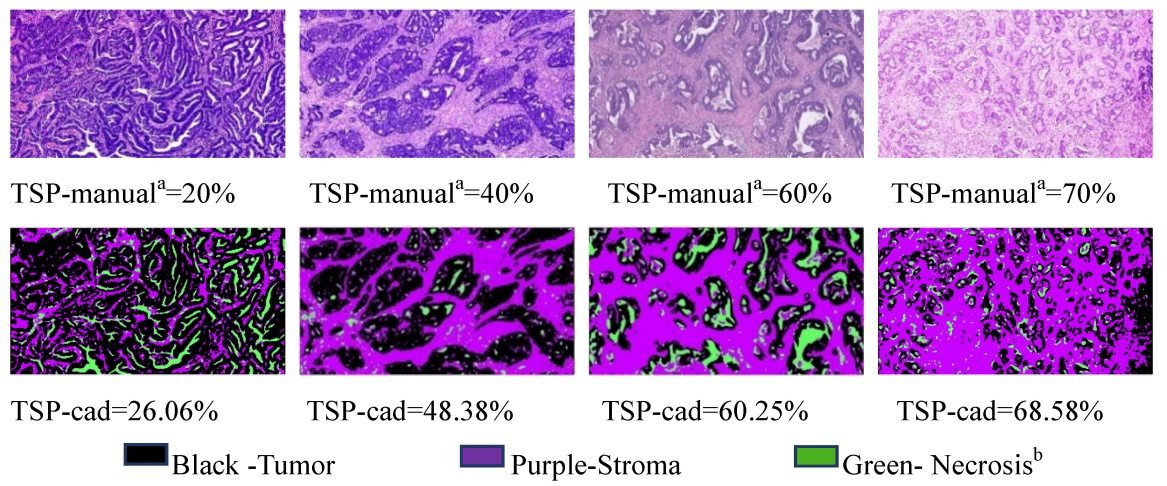
Representative matched H&E and segmented images of tumor (black), stroma (purple) and necrosis (green) using the pixel classifier algorithm in MATLAB. Top row: Stroma hot-spot rectangle, 100 ×150 μm across, selected by the observers for the assessment of TSP-manual and extracted with a diameter of x400 field for processing by the MATLAB Bottom row: The same regions in tissues was segmented by MATLAB to calculate TSP-cad. Pathologist^a^ consensus; Necrosis^b^ includes classes: fat, muscle, lymphocyte infiltrations, healthy epithelium, erythrocytes.

**Figure 4:**
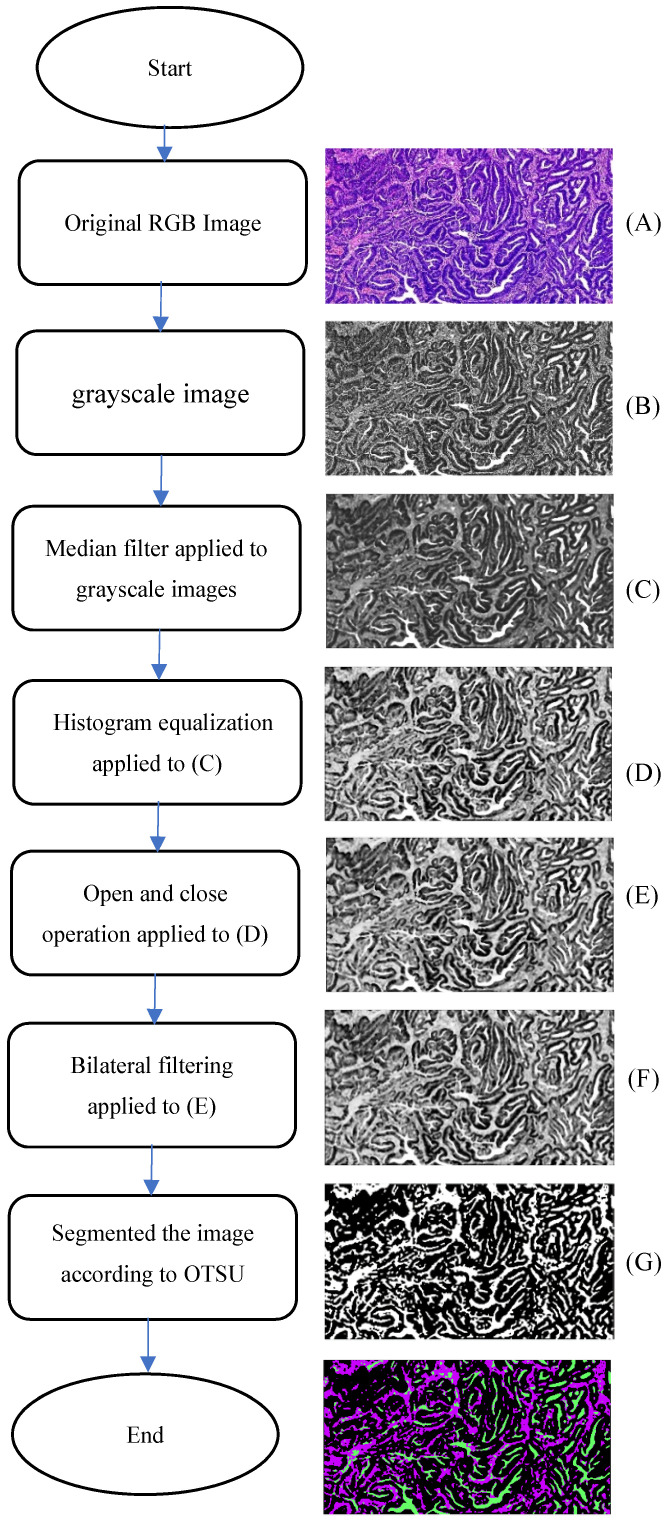
Schematic representation of the proposed algorithm.

Analysis of on postoperative H&E-stained images of CRC was performed by using the CAD systems and an open source software MATLAB was previously described in detail 11. Examples of representative TSP images generated by the pixel classifier in MATLAB is presented. The TSP images in the CAD systems included evaluating the ratio of tumor, stroma and necrosis (Fig. 3). The percentage of stroma was evaluated and was expressed and converted into WSIs. Here, TSP was the percentage of stroma based on the proposed CAD system with MATLAB software. We determined the gray image area as the stromal part of the WSI and calculated the stroma percentage on MATLAB software (Supplementary Fig. 1). Following background correction using two negative control sections, the images were quantified for calculating the gray or optimizing threshold (Fig. 4). Digital pathological images were collected in the Original Red-Green-Blue (RGB) style. In the proposed algorithm, all the images were converted into the gray scale images at the first step. Median filter 28 were adopted to remove the noises and artefact of the images and histogram equalization was used to enhance the contrast of the Digital pathological images. Then, the images after denoising and contrast enhancement were shown in Fig. 4D. Operating and closing operations and Bilateral filter 29 were adopted in order to further restrain the noise, enhance the contrast of the different structures, which made images smoother and lighter. OTSU image segmentation method 30 were used in the proposed pipeline, the best threshold t of this pipeline was set to be 0.82 after many preliminary experiments, which could be accurately identified as cellular parenchyma and intercellular substance.

### Statistical analyses and data analyses

We compared TSP-manual with TSP-cad as the prognostic factor in CRC in this study. Statistical analyses were performed using IBM SPSS Statistics software (version 24) and the figures were generated from GraphPad Prism 7 (version 7.02). Interobserver agreement on stroma percentage between the two pathologists and CAD system MATLAB software was analyzed by calculating intraclass correlation coefficients (ICC). Here, Pearson correlation analysis was adopted to compare the TSP evaluated by pathologists and the TSP from MATLAB of CAD system. We identified clinical factors associated with the TSP with binary logistic regression analysis. Univariable and multivariable Cox proportional hazard models were used to examine the significance of the clinical characteristics and stroma percentage as predictors of Overall Survival (OS). OS was defined as the time period between the date of primary surgery and the date of death from any cause or the date of last follow-up. Right sided tumors were defined as follows: coecum, colon ascendens, flexura hepatica, colon transversum and for left sided: flexura lienalis, colon descendens, colon sigmoideum, rectosigmoideum.

The two TSP-manual agreements between the two pathologists and CAD system MATLAB software were calculated using Cohen's Kappa (κ) on the dichotomized TSP values. Interobserver agreement was classified as “slight” (k =0.00-0.20), “fair” (k = 0.21-0.40), “moderate” (k = 0.41-0.60), “good” (k = 0.61-0.80), or “excellent” (k = 0.81-1.00). Paired and unpaired continuous variables were compared by Student's t test or the Mann-Whitney U test. Performance of the predictive TSP was evaluated from multiple dimensions. The linear trend χ^2^ score were used to assess discriminatory ability and monotonicity of each TSP. The likelihood-ratio (LR) χ^2^ test was used to assess homogeneity between TSP. Kaplan-Meier log rank analysis was used to examine the effect of TSP on OS. Univariate survival analysis for TSP-manual and TSP-cad used Cox proportional hazards regression to calculate 95% confidence intervals (95% CI) and hazard ratios (HRs). The linear trend ×2 test was used to analyze the correlation between TSP and clinicopathological features. P value <0.05 was considered statistically significant. All analyses were performed using IBM SPSS Statistics software (version 24).

## Results

### Clinicopathological data

**Figure 5:**
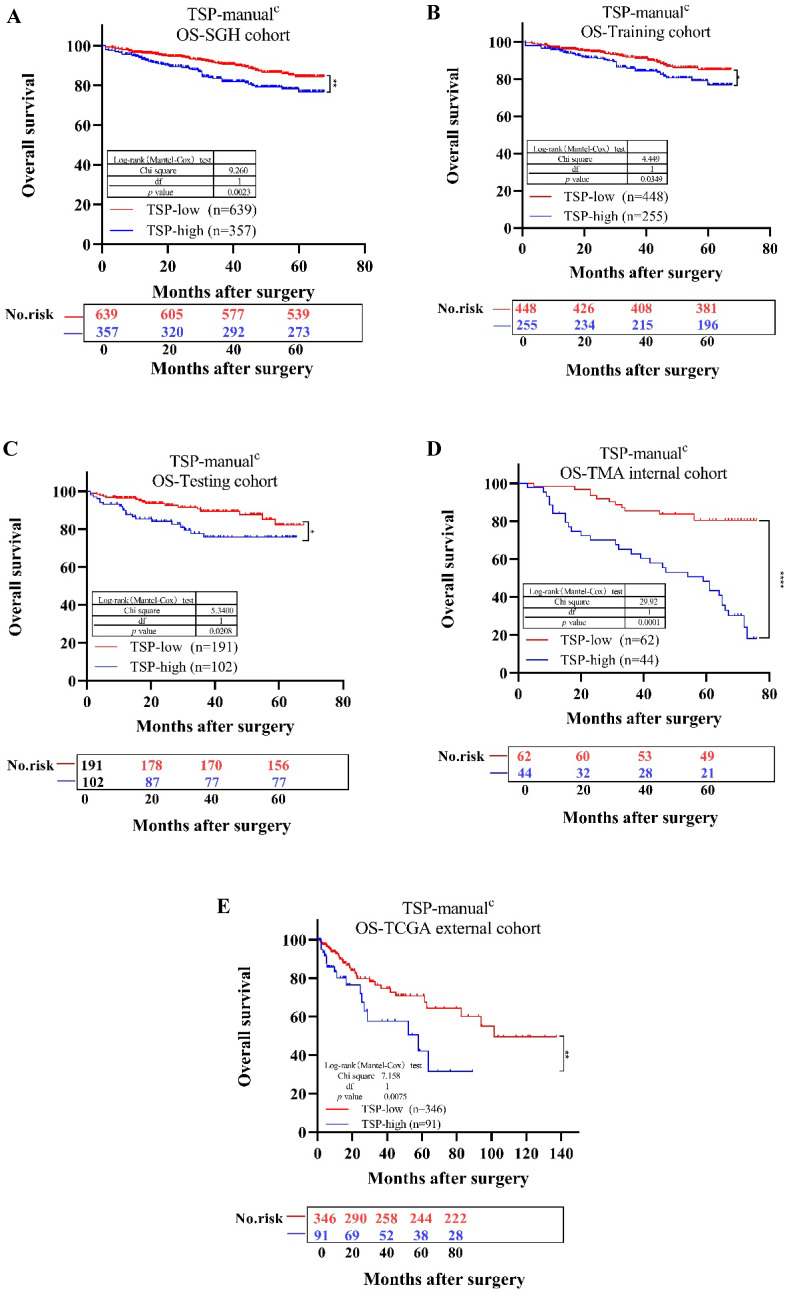
Kaplan-Meier survival analysis for overall survival of TSP-low verse TSP-high. Results for SGH cohort (A), Training cohort (B), Testing cohort (C), TMA internal cohort (D), TCGA external cohort (E) according to the TSP classifier stratified by clinicopathological risk factors.* P*-values were calculated by log-rank test.

We then determined whether TSP acted as a valuable variate for the survival and prognosis of CRC patients. First, for SGH cohort, the univariate analysis indicated that patients with TSP-high (TSP>50%) had poorer OS (P=0.0023) compared with those with TSP-low (TSP≤50%) (Fig. 5A). The TNM stages, depth of invasion, lymph node metastasis, nerve invasion, vessel invasion, differentiation status, tumor location and TSP were significantly related to OS, based on the univariate analysis (Table 2 and Supplementary Fig. 2). We conclude that TSP is of prognostic value for patients with either left and rectum tumor or right sided tumor, although for patients with a right sided tumor this is more evident 10. Second, according to the multivariate survival analysis based on the above factors in the Cox proportional hazards model, TSP-high (TSP>50%) was significantly related with OS (HR = 1.750; 95% CI, 1.214-2.523; P=0.003). These findings indicated that TSP-high (TSP>50%) was shown to have a poor prognosis in CRC.

### Performance measures

**Figure 6:**
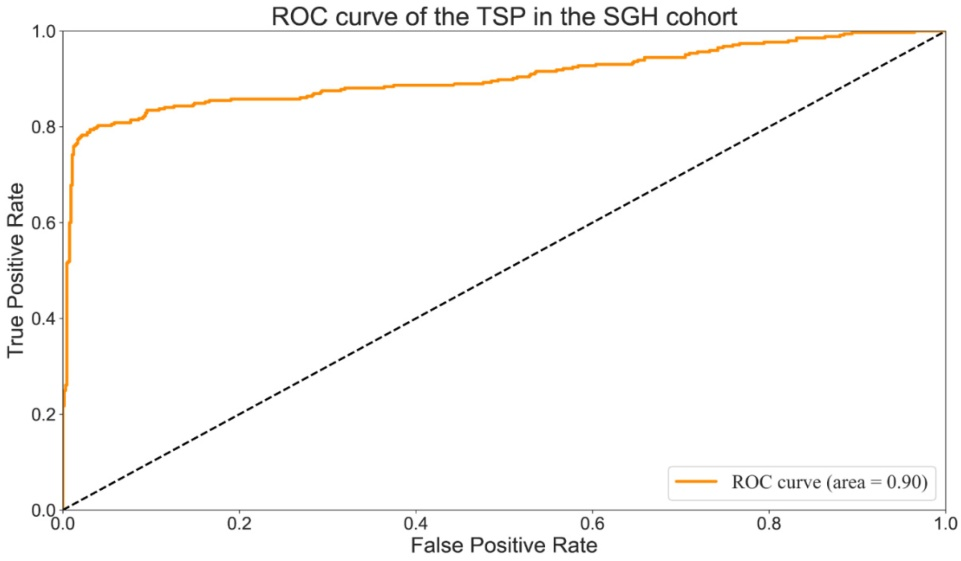
ROC curve of the TSP-manual^c^ in the SGH cohort.

A comparison between TSP drawn by a manual pathologist (TSP-manual) and those provided by the proposed method (TSP-cad) was carried out to assess the rapid multi-dynamic threshold algorithm performance in the segmentation of tumor stroma. True positive (TP) was the number of pixels existed both in TSP-manual and TSP-cad; false negative (FN) referred to all pixels which were failed to identified by the algorithm; false positive (FP) was all the pixels were identified automatically instead of manually. The segmentation performance which was evaluated by calculating the sensitivity, specificity, precision, defined as followed: We found that the overall accuracy was 90% (T Li,Y Yang), which showed improvement in researches by T Li et al. Values of per-class sensitivity, specificity, Precision are listed in Table 3. The Receiver Operating Characteristic Curve (ROC) 31 were shown in Fig. 6. The AUC (Area Under Curve) value was an important indicator for evaluating segmentation results because it was independent of the threshold. The AUC value of the TSP in the SGH cohort was 0.9 (Fig. 6), which had met the requirements of clinical screening.

### Agreement of TSP-manual and TSP-cad

**Figure 7:**
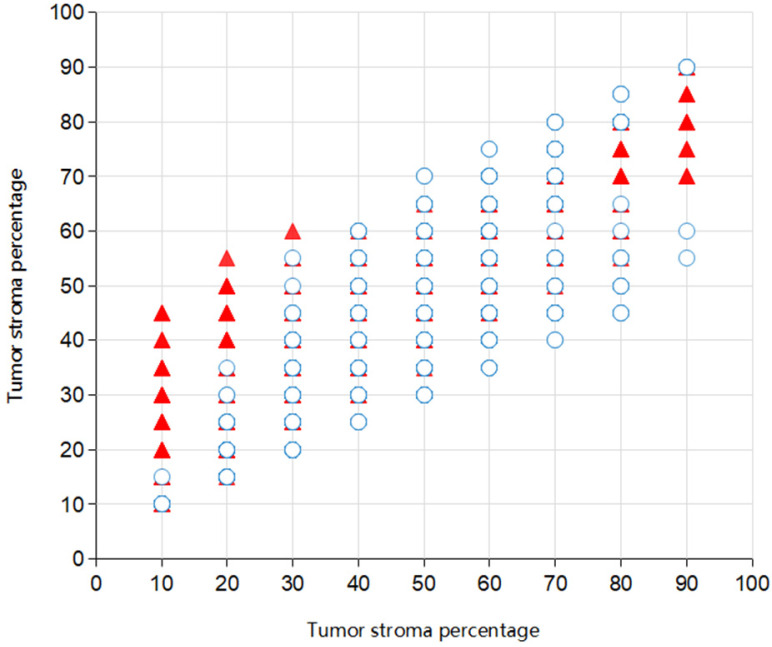
Scatter plot of TSP in 996 patients for Pathologist 1 and Pathologist 2. Overlapping parts indicated the consistent situation (321 in total) for the observers and the isolated one (24 in total) the inconsistent.

The ICC between the two pathologist for the TSP-manual was 0.545 (95% confidence interval (95% CI) 0.500-0.588). The picture depicted the co-occurrence of TSP scores evaluated by two pathologists (Fig. 7). The ICC's between TSP-cad and TSP-manual were 0.822 (95% CI 0.801-0.842). For the SGH cohort, according to the 50% cut-off value described previously, after dichotomizing the TSP visual object, a moderate agreement was found between the two pathologist (k=0.509). It was observed that a relatively high consistency only existed between TSP-manual and TSP-cad (k=0.813). When median was used as the cut-off value of TSP-cad, the consistency was slightly improved (k=0.552).The results were TSP-cad(median)≤41.30% (TSP-low), TSP-cad(median) >41.3%(TSP-high). The pathologist divided the patients into TSP-low group or TSP-high group. These results were shown in Table 4 and Table 5.

Using the same cut-off value of TSP-cad, for the Training cohort, the kappa between the TSP-manual and TSP-cad(50%) was 0.827, the kappa between the TSP-manual and TSP-cad(median) was 0.543 (Supplementary Table 3). For the testing cohort, the kappa between the TSP-manual and TSP-cad(50%) was 0.776, the kappa between the TSP-manual and TSP-cad(median) was 0.575 (Supplementary Table 4).

### Prognostic analyses

The established cut-off values at TSP were TSP-low≤50% and TSP-high>50%. In the SGH cohort, according to these thresholds, 357 cases (35.84%) were classified as TSP-high, 639 cases (64.16%) were classified as TSP-low. In the SGH cohort, a TSP-high was associated with TNM stage and lymph node metastasis in comparison to TSP-low (p=0.024 and p=0.035, Table 1, Table 6). In the TMA cohort, a TSP-high was associated with TNM stage and lymph node metastasis in comparison to TSP-low (p<0.001 and p<0.001, Table S1, Table 6). In the TCGA cohort, a TSP-high was associated with TNM stage and lymph node metastasis in comparison to TSP-low (p=0.024 and p=0.028, Supplementary Table 1, Table 6).

A further Kaplan-Meier analysis in the overall patient population showed a significant adverse OS for patients with a TSP-high and TSP-low. In the SGH cohort, the 5-year survival rate was separately 84.37% in patients with TSP-low and 76.69% in patients with TSP-high. Thereby, For the Training cohort, in the overall population (n=703), 255 patients (36.27%) were assigned to TSP-high group, 448 patients (63.73%) to the TSP-low group. For the Testing cohort, in the overall population (n=293), 102 patients (34.81%) were assigned to TSP-high group, 191 patients (65.19%) to the TSP-low group. A TSP-high was associated with TNM stage and worse OS in comparison to the TSP-low group (p=0.03 and p=0.016, Table 6 and Fig. 5C). The performance of each TSP method was assessed and summarized in Supplementary Table 5. In SGH cohort, TSP-cad(median) showed higher Linear trend χ^2^ score (OS: 13.445), higher LR test (OS:13.673) compared to the TSP-manual Linear trend χ2 score (OS: 12.025) LR test (OS: 11.601) and TSP-cad(50%) Linear trend χ^2^ score (OS: 10.953) LR test (OS: 10.3).

For TSP-manual, there was a significantly lower OS in the TSP-high compared to the TSP-low group in the SGH cohort (p=0.0023, Fig. 5A). The same outcomes were seen in the Training, Testing, TMA internal, TCGA external cohort (p=0.0349, p=0.0208, p<0.0001, p=0.0075, Fig. 5, respectively). Similarly, Due to we do not evaluate TSP using MATLAB of CAD system in the TMA and TCGA cohort, thus we lack of TSP-cad data in TMA and TCGA cohort. For TSP-cad(50%), the same outcome was seen in the SGH, Training, Testing cohort(p=0.0068, p=0.0305, p=0.0897, respectively), meanwhile, TSP-cad(median) had the same results ( p=0.002, p=0.0165, p=0.0469, respectively) (Supplementary Fig. 3).

In the univariate analysis, all methods for evaluating the TSP were found to be prognostic for OS in the all cohorts. For the SGH cohort: TSP-manual HR = 1.75 (95% CI 1.214-2.523); TSP-cad(50%) HR = 1.659 (95% CI 1.145-2.405); and TSP-cad(median) HR = 1.823 (95% CI 1.239-2.683) (Table 2). For the Training cohort: TSP-manual HR = 1.61 (95% CI 1.030-2.517); TSP-cad(50%) HR = 1.638 (95% CI 1.042-2.573) and TSP-cad(median) HR = 1.759 (95% CI 1.101-2.809) (Supplementary Table 6). For the Testing cohort: TSP-manual HR = 1.75 (95% CI 1.124-2.523); TSP-cad(50%) HR = 1.659 (95% CI 1.145-2.405); and TSP-cad(median) HR = 1.823 (95% CI 1.239-2.683) (Supplementary Table 7). Whether Training or Testing cohort, the result that TSP-cad(median) hazard ratio was slightly higher hazard ratio than TSP-manual, we found that we had the same results as others^27^,^32^. Thus, we concluded that the optimal cut-off value for TSP-cad(median) should be lower than previous internationally recognized cut-off of 50%^10^.

For multivariate analysis, the TSP-cad whether TSP-cad(50%) or TSP-cad(median), was found independent prognostic of vessel invasion and tumor location. For OS, In SGH cohort, TSP-manual HR = 1.516 (95% CI 1.048-2.193); TSP-cad(50%) HR = 1.452 (95% CI 0.999-2.111) and TSP-cad(median) HR=1.608 (95% CI 1.089-2.375) (Table 2).

## Discussion

Accurate assessment was essential for choosing the appropriate treatment. In routine clinical practice, the TNM staging system was a key prognostic determinant in CRC for oncologists and patients. However, the patients with the same cancer stage had different clinical outcomes, which indicated that the current staging system was not sufficient to predict prognosis. In this study, the CRC patients with high TSP was of poor prognosis based on the H&E-stained sections. There are differences between the assessments from pathologists' visual evaluation, which was not quite feasible to adopt widely 21. Thus we established a threshold algorithm for gray Image, which had acted as a powerful tool different from manual naked eye assessment. Our threshold algorithm could accurately quantify the percentage of tumor stroma area, which greatly improved its forecasting accuracy. In addition, in predicting the survival time, the kaplan-Meier log rank analysis suggested that the outcomes of our threshold algorithm were similar to those of current TNM stage. Although TNM staging was crucial to assess prognosis and establish a treatment strategy, the staging is performed mainly on the basis of anatomical information. In contrast, the TSP-cad threshold algorithm could improve the accuracy of prognosis. Application of TSP-manual with H&E-stained sections might be a straightforward and clinically applicable procedure. In our study, the combination of CAD systems and TSP assessment in different gray sections had a better prognostic value than TSP-manual assessment. These results suggested that the TSP-cad reinforced the prognostic competence of TNM stage, thereby adding more prognostic value to TNM staging. These results suggested that threshold algorithm of the TSP-cad might become a new constituent of the classification of CRC.

Although TSP assessment not a standard component of therapies for patients with CRC, previous reports indicated that TSP could improve their outcomes. The evaluation of the entire tissue sample was vital for CRC prognosis where the tumor stroma was determined as parts in between the TSP-low and the TSP-high. For practical reasons, pathologists typically examined representative regions in each slide before they came up with a prognostic decision (i.e., sampling). However, sampling might lead to prognostic errors, particularly in tumors with heterogeneity. Since the computer analyzed the ROI, it could potentially assist the pathologist in inconsistency in identifying TSP-low or TSP-high regions from pathological slides. This could have an important effect on the prognostic decision and hence could help to reducing the intra- and inter-reader variability.

Our independent tests showed that TSP-cad could be well evaluated by threshold algorithm for Gray Image. The kappa value of TSP-cad(median) (kappa=0.552) was slightly higher than TSP-manual (kappa=0.509). The TSP-cad(median) served as an independent prognostic factor for OS in the testing, training, TMA internal, TCGA external cohorts, respectively. The prognostic value of the TSP-cad(median) was compared to the assessed in consensus by two pathologists for OS both in the univariate and multivariate analysis.

Our study was to investigate and quantify the rapid multi-dynamic threshold algorithm approaches to possibly add prognostic significance to the TSP. Furthermore, a mild consistency was found in the assessment from two pathologists. On the whole, our TSP-manual value was lower than that was recognized by the TSP-cad. The main reason was that our threshold algorithm could well identify necrotic blank areas, which concluded that the mean percentage of necrotic areas was 12.025%. Fortunately, TSP-manual and TSP-cad(median) were also found as an independent prognostic factor in the SGH and Testing cohorts. We found TSP-cad(median) (HR=1.823 95%, CI 1.239-2.683) had slightly higher HR and wider CI than TSP-manual (HR=1.75,95% CI 1.214-2.523). As is shown in Supplementary Table 5, we observed that TSP-cad(median) showed higher Linear trend χ^2^ score compared to the TSP-manual and TSP-cad(50%), indicating that TSP-cad(median) had excellent discriminatory power in predicting prognosis of distinct cohort.

A similar recent study showed that stromal value cut-point of 33% is slightly less than the previous internationally recognized cut-point of 50% in Triple Negative and Luminal Breast Cancer 31. The discrepancy between pathologists and CAD system was also described between the cut-point for CAD analysis TSP assessment 65.47% versus 50% pathologists visual assessment in rectal cancer 11. The percentage difference in cut-point suggests there may be a common discrepancy between pathologists versus CAD when assessing a tumor pathology image. For the univariate and multivariate analysis, our research showed that the cut-off value for TSP-cad(median) in CRC was 41.3%. Interestingly our TSP cut-point is similar to cut-off value 48.8% of Zhao 27. Our results are both less than cut-point of 50% in CRC. Due to tumor heterogeneity, TSP varies greatly among patients with different tumors, and obvious interstitial fibrosis is one of the important features of pancreatic ductal adenocarcinoma, so cut-off may be inconsistent among different tumor types. Thus, there are reasons to believe that the optimal cut-off is less than previous internationally recognized cut-off of 50% in CRC, this also proves that our rapid multi-dynamic algorithm is highly accurate and practical. In addition, we did not separate the left colon and rectum into different groups. To the best of our knowledge, our study showed for the first time that we put the left colon and rectum together by using the threshold algorithm. In order to swiftly identify the TSP of the patient's postoperative pathological section, the rapid multi-dynamic method was applied into the future assessment methods of CRC.

In this article, we came up with an adaptive algorithm for the automatic discrimination between tumor and stroma percentage in CRC. To the best of our knowledge, the proposed method was a rapid multi-dynamic algorithm for the segmentation of stroma percentage in H&E-stained images of colorectal tissue. Also, TSP was firstly evaluated to verify its relevance to clinical related endpoints, and thus was acknowledged as a future biomarker in this setting. The algorithm was tested on 996 H&E images with high variation of staining intensities (Fig. 2). High segmentation performances were obtained for each image of the data cohort. Being a rapid multi-dynamic threshold algorithm, this algorithm could be used in future studies as a starting point to realize reliable systems for the tumor microenvironment and diagnosis. Another possible application of the threshold algorithm was to evaluate tumor's response to neoadjuvant therapy by comparing the relative proportion of percentage of tumor and the percentage of tumor stroma in the tumor stroma before and after chemotherapy. We analyzed the TSP of primary tumors from diagnostic biopsies and surgical resections. In surgically resected primary tumors, TSP low was detected in 47% of patients, a proportion number which was similar to the results in the literature. Our results demonstrate that tumors with low TSP had a significantly higher risk of vascular and neural invasion, along with T and N tumors, compared with tumors with high TSP. These observations confirmed statements in previous literature. Our research group was currently working on an extension of this algorithm for the semiautomatic quantification of the biomarkers expressed by stroma ingredient (e.g. Tumor-infiltrating lymphocytes, Tumor-associated macrophages, Cancer-associated fibroblasts) near the tumor boundary. Synchronously, we were carrying out large-scale promotion and verification of CRC data in five domestic hospitals.

To sum up, pathological pictures were digitally analyzed by using readily, available and free image processing and analysis software MALTAB. Our idea was to recalibrate with the software after the ratio of TSP was initially determined by the pathologist. In order to reduce individual differences in TSP judgment. By better understanding the role of the tumor stroma in CRC, our results showed that the rapid multi-dynamic algorithm could be a good prognostic information judgment. However, the results of a large population-based cohort study had not been widely reported. Although TSP hadn't been applied into TNM staging, it was believed to turn into a precise and customized therapy in the future. In future outside generalization validations, we continued to optimize this threshold grayscale image thresholding algorithm. We have reasons to believe that our study will be used on a large scale clinically in the future.

In conclusion, we find that in both TSP-cad(50%) and TSP-cad(median), multivariate analysis showed the TSP-cad was an independent prognostic factor for the vessel invasion and tumor location. Fortunately, TSP-manual and TSP-cad were also found independent prognostic factor in all the cohorts. It was found that TSP-cad had slightly higher HR and wider CI than TSP-manual and TSP-cad(median) showed higher Linear trend χ2 score compared to the TSP-manual and TSP-cad(50%). The optimal cut-off is less than previous internationally recognized cut-off of 50% in CRC. Thus, the current study demonstrates that rapid multi-dynamic threshold Algorithm for Gray Image counting of TSP using our proposed method on MATLAB software showed a higher accuracy than manual evaluation by pathologists.

## Supplementary Material

Supplementary figures and tables.

## Figures and Tables

**Table 1:** Clinicopathological data for SGH cohort in relation to TSP

Variable	TSP-manual				TSP-cad(50%)				TSP-cad(median)			
	N	TSP-low (%)	TSP-high (%)	p value	N	TSP-low (%)	TSP-high (%)	P value	N	TSP-low (%)	TSP-high (%)	P value
**Age (years)**				0.815				0.898				0.340
<65	461	294(46.0%)	167(46.8%)		461	330(46.4%)	131(46.0%)		461	238(47.8%)	223(44.8%)	
≥65	535	345(54.0%)	190(53.2%)		535	381(53.6%)	154(54.0%)		535	260(52.2%)	275(55.2%)	
**Gender**				0.458				0.462				0.797
Male	576	364(57.0%)	212(59.4%)		576	406(57.1%)	170(59.6%)		576	290(58.2%)	286(57.4%)	
Female	420	275(43.0%)	145(40.6%)		420	305(42.9%)	115(40.4%)		420	208(41.8%)	212(42.6%)	
**TNM Stage**				0.024*				0.025*				0.004*
I+II	610	408(63.8%)	202(56.6%)		610	451(63.4%)	159(55.8%)		610	327(65.7%)	283(56.8%)	
III+IV	386	231(36.2%)	155(43.4%)		386	260(36.6%)	126(44.2%)		386	171(34.3%)	215(43.2%)	
**Depth of invasion**			0.326				0.397				0.299	
T1+T2	195	131(20.5%)	64(17.9%)		195	144(20.3%)	51(17.9%)		195	104(20.9%)	91(18.3%)	
T3+T4	801	508(79.5%)	293(82.1%)		801	567(79.7%)	234(82.1%)		801	394(79.1%)	407(81.7%)	
**LN metastasis**			0.035*				0.035*				0.005*	
N0	607	405(63.4%)	202(56.6%)		607	448(63.0%)	159(55.8%)		607	325(65.3%)	282(56.6%)	
N1+N2	389	234(36.6%0	155(43.4%)		389	263(37.0%)	126(44.2%)		389	173(34.7%)	216(43.4%)	
**Nerve invasion**				0.558				0.382				0.277
NO	739	478(74.8%)	261(73.1%)		739	533(75.0%)	206(72.3%)		739	377(75.7%)	362(72.7%)	
YES	257	161(25.2%)	96(26.9%)		257	178(25.0%)	79(27.7%)		257	121(24.3%)	136(27.3%)	
**Vessel invasion**			0.408				0.261				0.453	
NO	683	444(69.5%)	239(66.9%)		683	495(69.6%)	188(66.0%)		683	347(69.7%)	336(67.5%)	
YES	313	195(30.5%)	118(31.1%)		313	216(30.4%)	97(34.0%)		313	151(30.3%)	162(32.5%)	
**Differentiation**				0.122				0.240				0.307
Well	85	48(7.5%)	37(10.4%)		85	56(7.9%)	29(10.2%)		85	38(7.6%)	47(9.4%)	
Moderate+poor	911	591(92.5%)	320(89.6%)		911	655(92.1%)	256(89.8%)		911	460(92.4%)	451(90.6%)	
**Tumor size**				0.476				0.174				1.000
<5 cm	620	403(63.1%)	217(60.8%)		620	452(63.6%)	168(58.9%)		620	310(62.2%)	310(62.2%)	
≥5 cm	376	236(63.9%)	140(39.2%)		376	259(36.4%)	117(41.1%)		376	188(37.8%)	188(37.8%)	
**Tumor location**			0.762				0.481				0.418	
Right	326	207(32.4%)	119(33.3%)		326	228(32.1%)	98(34.4%)		326	169(33.9%)	157(31.5%)	
Left and rectal	670	432(67.7%)	238(66.7%)		670	483(67.9%)	187(65.6%)		670	329(66.1%)	341(68.5%)	

Note. P-value is derived from the univariable association analyses between each of the clinicopathological variables and treatment response. The clinical characters were the data from the initial diagnosis. Abbreviations: LN metastasis: Lymph node metastasis. *P < 0.05.

**Table 2:** Uni- and multivariate Cox regression analysis for overall survival in the SGH cohort

	Univariate analysis		Multivariate analysis					
			TSP-manual^a^		TSP-cad(50%)		TSP-cad(median)	
	HR	95%CI	HR	95%CI	HR	95%CI	HR	95%CI
**Age (years)**								
<65	1							
≥65	2.368	1.646-3.407						
**Gender**								
Male	1							
Female	0.613	0.413-0.909						
**TNM Stage**								
I+II	1		1		1		1	
III+IV	3.54	2.404-5.212*	1.176	0.457-3.023	1.198	0.473-3.037	1.241	0.501-3.075
**Depth of invasion**								
T1+T2	1		1		1		1	
T3+T4	2.878	1.504-5.506*	1.401	0.771-2.762	1.402	0.711-2.765	1.383	0.701-2.729
**LN metastasis**								
N0	1		1		1		1	
N1+N2	3.724	2.516-5.512*	2.372	0.908-6.197	2.349	0.912-6.049	2.228	0.884-5.615
**Nerve invasion**								
NO	1		1		1		1	
YES	2.298	1.575-3.353*	1.397	0.917-2.130	1.421	0.943-2.164	1.413	0.928-2.152
**Vessel invasion**								
NO	1		1		1		1	
YES	2.684	1.858-3.875*	1.575	1.034-2.400*	1.565	1.028-2.383*	1.574	1.034-2.398*
**Differentiation status**								
Well	1		1		1		1	
Moderate+poor	2.574	1.050-6.312*	1.964	0.795-4.851	1.986	0.804-4.910	1.957	0.799-4.877
**Tumor size**								
<5 cm	1							
≥5 cm	1.287	0.889-1.862						
**Tumor location**								
Right	1		1		1		1	
Left and rectal	0.502	0.348-0.723*	0.498	0.342-0.725*	0.492	0.329-0.717*	0.486	0.334-0.707*
**TSP-manual^a^**								
TSP-low	1		1					
TSP-high	1.75	1.214-2.523*	1.516	1.048-2.193*				
**TSP-cad(50%)**								
TSP-low	1				1			
TSP-high	1.659	1.145-2.405*			1.452	0.999-2.111*^b^		
**TSP-cad(median)**								
TSP-low	1						1	
TSP-high	1.823	1.239-2.683*					1.608	1.089-2.375*

^a^Pathology consensus; ^b^Duo to P-values is 0.051,which is near to 0.05, and shall be considered to siginificant results (P<0.05); Abbreviations: HR: hazard ratio; CI: confidence interval; LN metastasis:Lymph node metastasis. *P < 0.05.

**Table 3:** Comparison between TSP-manual and TSP-cad in the segmentation of tumor stroma

	TN	TP	FN	TP	sensitivity	specificity	Precision
Value	634	77	5	280	98.25%	89.17%	78.43%

**Table 4:** Cross-tabulation of Pathologist 1 versus Pathologist 2 after dichotomisation in SGH cohort

	Pathologist 1		
	TSP-low	TSP-high	Total
**Pathologist 2,** K=0.509			
TSP-low	621	18	639
TSP-high	184	173	357
Total	805	191	996

**Table 5:** Cross-tabulation of TSP-manual versus TSP-cad after dichotomisation in the SGH cohort

	TSP-manual^a^		
	TSP-low	TSP-high	Total
**TSP-cad (50%),** K=0.813			
TSP-low	634	77	711
TSP-high	5	280	285
Total	639	357	996
**TSP-cad (median),** K=0.552			
TSP-low	457	41	498
TSP-high	182	316	498
Total	639	357	996

**Table 6:** TSP-high was associated with TNM stage and lymph node metastasis in comparison to TSP-low in SGH, Training, Testing, TMA internal, TCGA external cohort

	TSP-manual	TSP-cad (50%)	TSP-cad(median)
	p value		
SGH cohort	0.024*	0.025*	0.004*
Training cohort	0.423	0.323	0.052
**TNM stage**			
Testing cohort	0.003*	0.008*	0.023*
TMA cohort	0.001*	NA	0.001*
TCGA cohort	0.024*	NA	0.032*
SGH cohort	0.035*	0.035*	0.005*
Training cohort	0.367	0.258	0.063
**Lymph node**			
Testing cohort	0.012*	0.026*	0.023*
**Metastasis**			
TMA cohort	0.001*	NA	0.002*
TCGA cohort	0.028*	NA	0.036*

Abbreviations: NA, not applicable. Due to we do not evaluate TSP using MATLAB of CAD system in the TMA and TCGA cohort, thus we lack of TSP-cad(50%)data in TMA and TCGA cohort. *P < 0.05.
