# Supplementary material for: Rapid multi-dynamic algorithm for gray image analysis of the stroma percentage on colorectal cancer
Source: J Cancer. 2021 Jun 1;12(15):4561–73. doi: 10.7150/jca.58887 (PMC8210572; doi:10.7150/jca.58887)
Supplement: Supplementary file 1 — Supplementary figures and tables. [file jcav12p4561s1.pdf]

# Materials Figure for

# Rapid multi-dynamic algorithm for gray image analysis of the stroma percentage on colorectal cancer

**Supplementary Figure**

A B

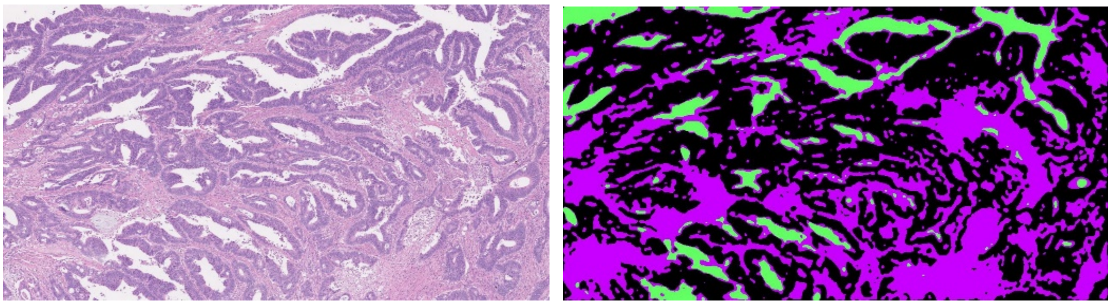

Green- Necrosis (num1) Black -Tumor (num2) Purple-Stroma (num3)

**Supplementary Figure 1.** The regions with tissues segmented by the MATLAB for the calculation of TSP-cad.

(A)Original Red-Green-Blue(RGB) style (B)Image was annotated using tumor, stroma and necrosis labels.

The result was num1= 0.0863 num2= 0.5459 num3=0.368

we considered the tumor and stroma proportions to be complementary.

TSP=Stroma/(Tumor+Stroma)*100%=num3/(num2+num3)*100%=0.368/(0.5459+0.368) *100%=40.27%

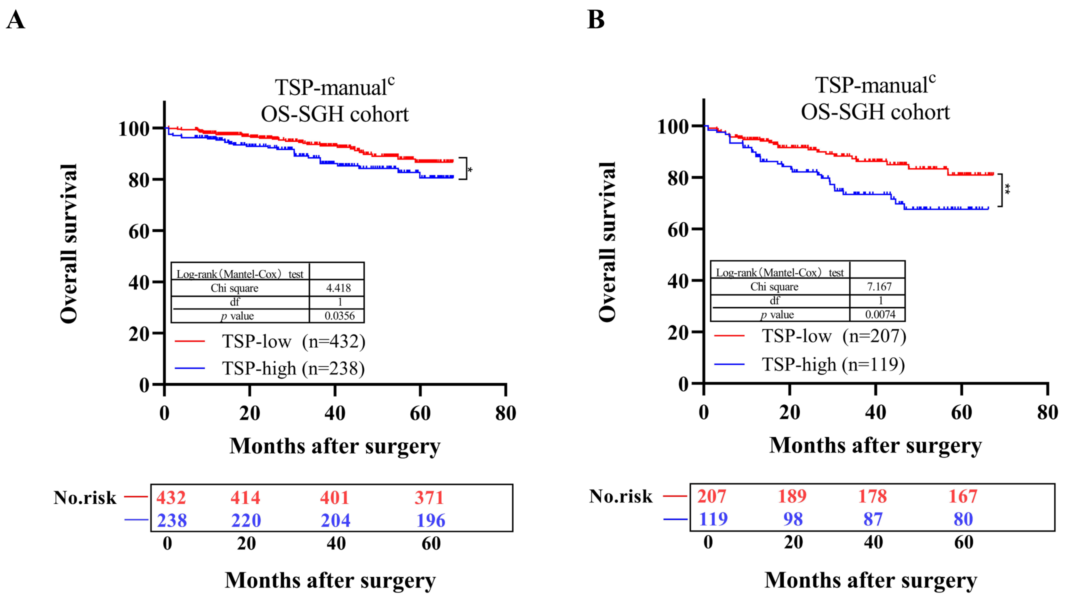


**Supplementary Figure 2.** Kaplan-Meier survival analysis curves of SGH chort for TSP-low verse TSP-high. (A) represent the OS of the left sided and rectum tumors (B) represent the OS of the right sided tumors.

A

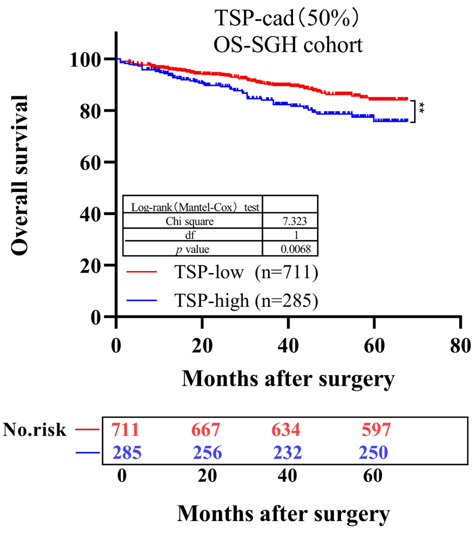
TSP-cad（50%）
OS-SGH cohort
Overall survival
Log-rank (Mantel-Cox) test
Chi square 7.323
df 1
p value 0.0068
TSP-low (n=711)
TSP-high (n=285)
**
Months after surgery
No.risk
711 667 634 597
285 256 232 250
Months after surgery

B

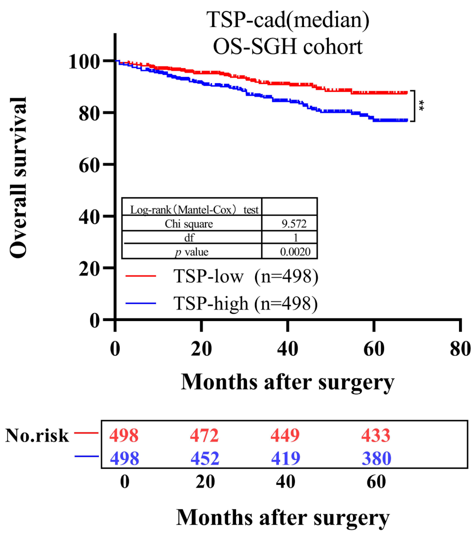
TSP-cad(median)
OS-SGH cohort
Overall survival
Log-rank (Mantel-Cox) test
Chi square 9.572
df 1
p value 0.0020
TSP-low (n=498)
TSP-high (n=498)
**
Months after surgery
No.risk
498 472 449 433
498 452 419 380
Months after surgery

C

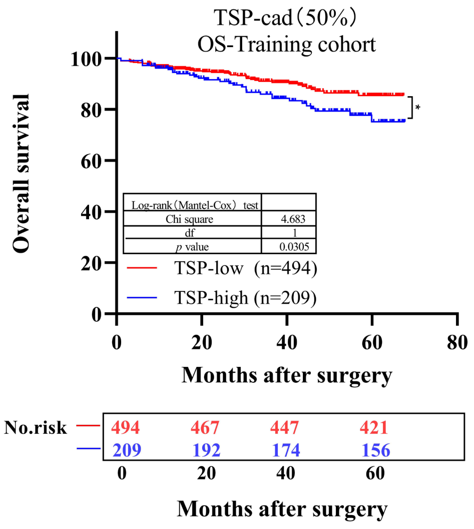
TSP-cad（50%）
OS-Training cohort
Overall survival
Log-rank (Mantel-Cox) test
Chi square 4.683
df 1
p value 0.0305
TSP-low (n=494)
TSP-high (n=209)
*
Months after surgery
No.risk
494 467 447 421
209 192 174 156
Months after surgery

D

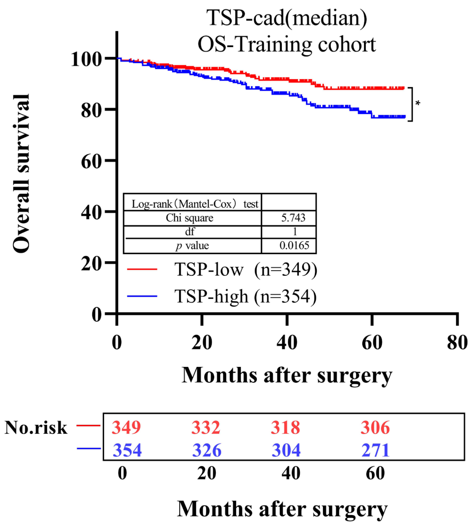
TSP-cad(median)
OS-Training cohort
Overall survival
Log-rank (Mantel-Cox) test
Chi square 5.743
df 1
p value 0.0165
TSP-low (n=349)
TSP-high (n=354)
*
Months after surgery
No.risk
349 332 318 306
354 326 304 271
Months after surgery

E

F

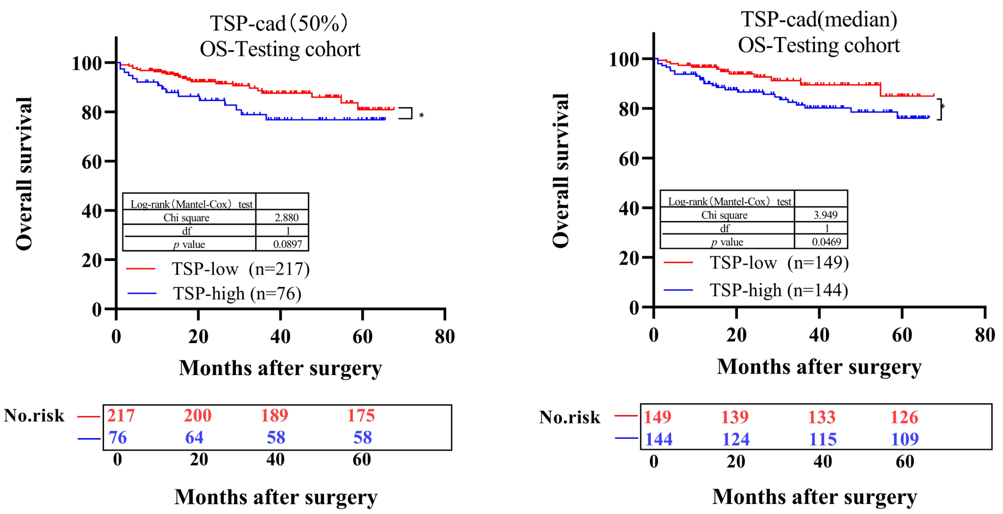


**Supplementary Figure 3.** Kaplan-Meier survival analysis for overall survival of TSP-low verse TSP-high. Results for SGH cohort (A,B), Training cohort (C,D), Testing cohort (E,F), according to the TSP-cad(50%) and TSP-cad(medium) classifier stratified by clinicopathological risk factors. *P*-values were calculated by log-rank test.

# Materials Table for

# Rapid multi-dynamic algorithm for gray image analysis of the stroma percentage on colorectal cancer

**Supplementary Table**

**Supplementary Table 1** Clinicopathological data for TMA cohort in relation to TSP-manual

| Variable | TSP-manual[a] | | | |
|---|---|---|---|---|
| | N | TSP-low(%) | TSP-high(%) | p value |
| Age(years) | | | | 0.057 |
| <65 | 55 | 37(59.7%) | 18(40.9%) | |
| ≥65 | 51 | 25(40.3%) | 26(59.1%) | |
| Gender | | | | 0.947 |
| Male | 55 | 32(51.6%) | 23(52.3%) | |
| Female | 51 | 30(48.4%) | 21(47.7%) | |
| TNM Stage | | | | $< 0.001^{*}$ |
| I+II | 51 | 41(66.1%) | 10(22.7%) | |
| III+IV | 55 | 21(33.9%) | 34(77.3%) | |
| Depth of invasion | | | | 0.991 |
| T1+T2 | 12 | 7(11.3%) | 5(11.4%) | |
| T3+T4 | 94 | 55(88.7%0 | 39(88.6%) | |
| Lymph node metastasis | | | | $< 0.001^{*}$ |
| N0 | 49 | 39(62.9%) | 10(22.7%) | |
| N1+N2 | 57 | 23(37.1%) | 34(77.3%) | |
| Nerve invasion | | | | 0.720 |
| NO | 79 | 47(75.8%) | 32(73.7%) | |
| YES | 27 | 15(24.2%) | 12(27.3%) | |
| Vessel invasion | | | | 0.061 |
| NO | 66 | 34(54.8%) | 32(72.7%) | |
| YES | 40 | 28(45.2%) | 12(27.3%) | |

| | | | | |
|---|---|---|---|---|
| Differentiation | | | | 0.954 |
| Well | 19 | 11(17.7%) | 8(18.2%) | |
| Moderate+poor | 87 | 51(82.3%) | 36(81.8%) | |
| Tumor size | | | | 0.323 |
| <5cm | 59 | 37(59.7%) | 22(50.0%) | |
| ≥5cm | 47 | 25(40.3%) | 22(50.0%) | |
| Tumor location | | | | 0.107 |
| Right | 41 | 20(32.3%) | 21(47.7%) | |
| Left and rectal | 65 | 42(67.7%) | 23(52.3%) | |

*P < 0.05

**Supplementary Table 2** Clinicopathological data for TCGA cohort in relation to TSP-manual

| Variable | TSP- manual[a] | | | |
|---|---|---|---|---|
| | N | TSP-low(%) | TSP-high(%) | p value |
| Age(years) | | | | 0.697 |
| <65 | 200 | 160(46.2%) | 40(44.0%) | |
| ≥65 | 237 | 186(53.8%) | 51(56.0%) | |
| Gender | | | | 0.647 |
| Male | 235 | 188(54.3%) | 47(51.6%) | |
| Female | 202 | 158(45.7%) | 44(48.4%) | |
| TNM stage | | | | 0.024* |
| I+II | 238 | 198(57.2%) | 40(44.0%) | |
| III+IV | 199 | 148(42.8%) | 51(56.0%) | |
| Depth of invasion | | | | 0.021* |
| T1+T2 | 91 | 80(23.1%) | 11(12.1%) | |
| T3+T4 | 346 | 266(76.9%) | 80(87.9%) | |
| Lymph node metastasis | | | | 0.028* |
| N0 | 246 | 204(59.0%) | 42(46.2%) | |
| N1+N2 | 191 | 142(41.0%) | 49(53.8%) | |
| lymphatic invasion | | | | 0.237 |
| NO | 287 | 232(67.1%) | 55(60.4%) | |
| YES | 150 | 114(32.9%) | 36(39.6%) | |
| Vessel invasion | | | | 0.61 |
| NO | 340 | 271(78.3%) | 69(75.8%) | |
| YES | 97 | 75(21.7%) | 22(24.2%) | |

| | | | | |
|---|---|---|---|---|
| Tumor location | | | | 0.662 |
| Right | 169 | 132(38.2%) | 37(40.7%) | |
| Left and rectal | 268 | 214(61.8%) | 54(59.3%) | |

*P < 0.05

**Supplementary Table 3** Cross-tabulation of TSP-mannual versus TSP-cad after dichotomisation in the Training cohort

| | | TSP-manual | | |
|---|---|---|---|---|
| | | TSP-low | TSP-high | total |
| TSP-cad(50%) | TSP-low | 444 | 50 | 494 |
| K=0.827 | TSP-high | 4 | 205 | 209 |
| | total | 448 | 255 | 703 |
| TSP-cad(median) | TSP-low | 318 | 31 | 349 |
| K=0.543 | TSP-high | 130 | 224 | 354 |
| | total | 448 | 255 | 703 |

**Supplementary Table 4** Cross-tabulation of TSP-mannual versus TSP-cad after dichotomisation in the Testing cohort

| | | TSP-manual | | |
|---|---|---|---|---|
| | | TSP-low | TSP-high | total |
| TSP-cad(50%) | TSP-low | 190 | 27 | 217 |
| K=0.776 | TSP-high | 1 | 75 | 76 |
| | total | 191 | 102 | 293 |
| TSP-cad(median) | TSP-low | 139 | 10 | 149 |
| K=0.575 | TSP-high | 52 | 92 | 144 |
| | total | 191 | 102 | 293 |

**Supplementary Table 5** Predictive performance comparison among the different TSP in SGH cohort

| SGH cohort | Linear trend χ2 score | LR test |
|---|---|---|
| Over survival | | |
| TSP-manual | 12.025 | 11.601 |
| TSP-cad(50%) | 10.953 | 10.3 |
| TSP-cad(median) | 13.445 | 13.673 |

LR, likelihood ratio;

**Supplementary Table 6** Uni- and multivariate Cox regression analysis for overall survival in the Traning cohort

| | Unvariate analysis | | Multivariate analysis | | | | | |
|---|---|---|---|---|---|---|---|---|
| | | | TSP-manual | | TSP-cad(50%) | | TSP-cad(median) | |
| | HR | 95%CI | HR | 95%CI | HR | 95%CI | HR | 95%CI |
| Age(years) | | | | | | | | |
| <65 | 1 | | | | | | | |
| ≥65 | 3.508 | 1.804-5.183 | | | | | | |
| Gender | | | | | | | | |
| Male | 1 | | | | | | | |
| Female | 0.594 | 0.368-0.956 | | | | | | |
| TNM Stage | | | | | | | | |

| | | | | | | | | |
|---|---|---|---|---|---|---|---|---|
| I+II | 1 | | 1 | | 1 | | 1 | |
| III+IV | 3.695 | 2.291-5.959* | 1.818 | 0.563-5.869 | 1.822 | 0.571-5.811 | 1.755 | 0.569-5.411 |
| Depth of invasion | | | | | | | | |
| T1+T2 | 1 | | 1 | | 1 | | 1 | |
| T3+T4 | 2.966 | 1.364-6.452* | 1.576 | 0.697-3.564 | 1.556 | 0.687-3.524 | 1.574 | 0.696-3.561 |
| LN metastasis | | | | | | | | |
| N0 | 1 | | 1 | | 1 | | 1 | |
| N1+N2 | 3.584 | 2.223-5.779* | 1.497 | 0.464-4.830 | 1.494 | 0.469-4.759 | 1.516 | 0.494-4.653 |
| Nerve invasion | | | | | | | | |
| NO | 1 | | 1 | | 1 | | 1 | |
| YES | 2.165 | 1.359-3.450* | 1.191 | 0.713-1.992 | 1.21 | 0.724-2.021 | 1.217 | 0.729-2.031 |
| Vessel invasion | | | | | | | | |
| NO | 1 | | 1 | | 1 | | 1 | |
| YES | 3.004 | 1.913-4.716* | 1.981 | 1.190-3.298* | 1.98 | 1.189-3.295* | 1.965 | 1.182-3.267* |
| Differentiation status | | | | | | | | |
| Well | 1 | | | | | | | |
| Moderate+poor | 1.511 | 0.610-3.743 | | | | | | |
| Tumor size | | | | | | | | |
| <5cm | 1 | | | | | | | |
| ≥5cm | 1.059 | 0.670-1.675 | | | | | | |
| Tumor location | | | | | | | | |
| Right | 1 | | 1 | | 1 | | 1 | |
| Left and rectal | 0.564 | 0.360-0.884* | 0.581 | 0.366-0.992* | 0.575 | 0.363-0.913* | 0.567 | 0.358-0.898* |
| TSP-manual | | | | | | | | |
| TSP-low | 1 | | 1 | | | | | |
| TSP-high | 1.61 | 1.030-2.517* | 1.376 | 0.876-2.161* | | | | |
| TSP-cad(50%) | | | | | | | | |
| TSP-low | 1 | | | | 1 | | | |
| TSP-high | 1.638 | 1.042-2.573* | | | 1.413 | 0.908-2.256* | | |
| TSP-cad(median) | | | | | | | | |
| TSP-low | 1 | | | | | | 1 | |
| TSP-high | 1.759 | 1.101-2.809* | | | | | 1.539 | 0.960-2.468* |

Abbreviations: HR: hazard ratio; CI: confidence interval; LN metastasis:Lymph node metastasis

*$P < 0.05$

**Supplementary Table 7** Uni- and multivariate Cox regression analysis for overall survival in the Testing cohort

| | Unvariate nalysis | | Multivariate analysis | | | | | |
|---|---|---|---|---|---|---|---|---|
| | | | TSP-manual | | TSP-cad(50%) | | TSP-cad(median) | |
| | HR | 95%CI | HR | 95%CI | HR | 95%CI | HR | 95%CI |
| Age(years) | | | | | | | | |
| <65 | 1 | | | | | | | |
| ≥65 | 2.368 | 1.646-3.407 | | | | | | |
| Gender | | | | | | | | |
| Male | 1 | | | | | | | |
| Female | 0.613 | 0.413-0.909 | | | | | | |

| | | | | | | | | |
|---|---|---|---|---|---|---|---|---|
| TNM Stage | | | | | | | | |
| I+II | 1 | | 1 | | 1 | | 1 | |
| III+IV | 3.54 | 2.404-5.212* | 1.171 | 0.443-3.096 | 1.197 | 0.46-3.114 | 1.214 | 0.486-3.169 |
| Depth of invasion | | | | | | | | |
| T1+T2 | 1 | | 1 | | 1 | | 1 | |
| T3+T4 | 2.878 | 1.504-5.506* | 1.343 | 0.681-2.65 | 1.343 | 0.68-2.651 | 1.315 | 0.666-2.597 |
| LN metastasis | | | | | | | | |
| N0 | 1 | | 1 | | 1 | | 1 | |
| N1+N2 | 3.724 | 2.516-5.512* | 2.334 | 0.869-6.268 | 2.305 | 0.872-6.092 | 2.186 | 0.841-5.678 |
| Nerve invasion | | | | | | | | |
| NO | 1 | | 1 | | 1 | | 1 | |
| YES | 2.298 | 1.575-3.353* | 1.38 | 0.96-2.103 | 1.41 | 0.927-2.145 | 1.398 | 0.919-2.126 |
| Vessel invasion | | | | | | | | |
| NO | 1 | | 1 | | 1 | | 1 | |
| YES | 2.684 | 1.858-3.875* | 1.608 | 1.055-2.451* | 1.593 | 1.045-2.427* | 1.593 | 1.046-2.425* |
| Differentiation status | | | | | | | | |
| Well | 1 | | 1 | | 1 | | 1 | |
| Moderate+poor | 2.574 | 1.050-6.312* | 2.613 | 0.874-5.352 | 2.19 | 0.884-5.423 | 2.198 | 0.888-5.443 |
| Tumor size | | | | | | | | |
| <5cm | 1 | | | | | | | |
| ≥5cm | 1.287 | 0.889-1.862 | | | | | | |
| Tumor location | | | | | | | | |
| Right | 1 | | 1 | | 1 | | 1 | |
| Left and rectal | 0.502 | 0.348-0.723* | 0.467 | 0.320-0.682* | 0.461 | 0.316-0.673* | 0.456 | 0.312-0.664* |
| TSP-manual | | | | | | | | |
| TSP-low | 1 | | 1 | | | | | |
| TSP-high | 1.75 | 1.214-2.523* | 1.512 | 1.045-2.187* | | | | |
| TSP-cad(50%) | | | | | | | | |
| TSP-low | 1 | | | | 1 | | | |
| TSP-high | 1.659 | 1.145-2.405* | | | 1.443 | 0.993-2.097 | | |
| TSP-cad(median) | | | | | | | | |
| TSP-low | 1 | | | | | | 1 | |
| TSP-high | 1.823 | 1.239-2.683* | | | | | 1.632 | 1.105-2.410* |

Abbreviations: HR: hazard ratio; CI: confidence interval; LN metastasis:Lymph node metastasis

*P < 0.05
